# Does residential exposure to air pollutants influence mortality and cardiovascular morbidity of older people from primary care?

**DOI:** 10.1186/s12889-023-16166-w

**Published:** 2023-07-03

**Authors:** Maurizio Pietro D’Acquisto, Dietmar Krause, Renate Klaassen-Mielke, Matthias Trampisch, Hans Joachim Trampisch, Ulrike Trampisch, Henrik Rudolf

**Affiliations:** 1 Department of Medical Informatics, Biometry and Epidemiology, Ruhr University Bochum, Bochum, Germany; 2 Department of Geriatric Medicine, Marien Hospital Herne, Ruhr University Bochum, Herne, Germany; 3 Boehringer Ingelheim Pharma GmbH & Co. KG, Ingelheim Am Rhein, Germany; 4 Institute for Biostatistics and Informatics in Medicine and Ageing Research, Rostock University Medical Center, Rostock, Germany

**Keywords:** Particulate matter, Nitrogen dioxide, Air pollution, Mortality, Older people, Peripheral artery disease

## Abstract

**Background:**

Diseases affecting the cardiovascular system are the most common cause of death worldwide. In addition to classical risk factors of atherosclerosis, long-term exposure to particulate matter with particles of size up to 10 µm (PM10) in the atmosphere has become an increasing focus of scientific attention in recent decades. This study analyses the associations of residential-associated air pollutants exposure with all-cause mortality and cardiovascular morbidity of older patients in a primary care setting.

**Methods:**

The “German Epidemiological Trial on Ankle Brachial Index” (getABI) is a prospective cohort study that started in 2001 and included 6,880 primary care patients with a follow-up of 7 years. The PM10 and nitrogen dioxide (NO_2_) concentrations in the atmosphere are interpolated values from the study "Mapping of background air pollution at a fine spatial scale across the European Union". The primary outcome in this analysis is death of any cause, a secondary outcome is onset of PAD. Cox proportional hazards regression was used in a two-step modelling, the first step with basic adjustment only for age, sex, and one or more air pollutants, the second with additional risk factors.

**Results:**

A total of 6,819 getABI patients were included in this analysis. 1,243 of them died during the study period. The hazard ratio (HR) (1.218, 95%-confidence-interval (CI) 0.949–1.562) for the risk of death from any cause was elevated by 22% per 10 µg/m^3^ increase of PM10 in the fully adjusted model, although not statistically significant. Increased PM10 exposure in combination with the presence of PAD had a significantly increased risk (HR = 1.560, 95%-CI: 1.059-2.298) for this endpoint in the basic adjustment, but not in the fully adjusted model. 736 patients developed peripheral artery disease (PAD) during the course of the study. There was no association of air pollutants and the onset of PAD.

**Conclusions:**

Our analysis renders some hints for the impact of air pollutants (PM10, NO_2_, and proximity to major road) on mortality. Interaction of PAD with PM10 was found. There was no association of air pollutants and the onset of PAD.

**Trial registration:**

German Clinical Trials Register: DRKS00029733 (19/09/2022).

## Introduction

Diseases affecting the cardiovascular system are the most common cause of death worldwide. According to the World Health Organization (WHO), 17.9 million people died from cardiovascular diseases in 2016. Therefore, almost one in three deaths of the total number of people who died in 2016 (56.9 million deaths worldwide) was due to cardiovascular disease [1]. Therefore, the study of pathomechanisms, risk factors, and prevention as well as treatment options of cardiovascular diseases is in the focus of medical research.

The term cardiovascular disease encompasses several complex clinical pictures. These include coronary artery disease, cerebrovascular disease, and peripheral artery disease (PAD).

Basically, atherosclerosis is assumed to be the cause of most cardiovascular diseases. Besides classic risk factors such as age, sex, nicotine consumption, diabetes mellitus, elevated cholesterol levels, and arterial hypertension, there may be other risk factors, which are not taken into account in everyday clinical practice. In particular, long-term exposure to particulate matter with particles of size up to 10 µm (PM10) from diesel soot, combustion reactions, and reactions of harmful gases in the atmosphere have become an increasing focus of scientific attention in recent decades [2].

Several studies have documented that short-term as well as long-term exposure to PM10 have a significant effect on the incidence and outcome of coronary heart disease, cerebrovascular disease and pulmonary diseases [3–8]. Thus, PM10 may have an impact on overall mortality as well as cardiovascular morbidity.

Although the exact effect of PM10 is still unknown, scientists suspect that an inflammatory response occurs in tissues exposed to particulate matter. Oxidative stress and the direct, damaging effects of PM10 cause further changes in the tissue. This process manifests itself in increased inflammatory markers in the serum, elevated blood pressure, glucose tolerance disorders, and reduced lung function. Atherosclerotic processes also advance more rapidly by exposure to PM10 [3, 9].

Since the beginning of 2005, upper limits for PM10 were established in the EU, meaning that the PM10 concentration must not exceed the daily limit of 50 µg/m^3^ more than 35 times a year. In addition, the annual mean PM10 concentration must not exceed 40 µg/m^3^. An annual mean value for PM2.5 was introduced in 2008. It amounts to 25 µg/m^3^ [10]. In several studies, such as the Heinz Nixdorf Recall Study and the European Study of Cohorts for Air Pollution Effects (ESCAPE), it was shown that exposure–response relationships occur even below the EU limit values [3, 4, 11]. In 2021, the WHO had called for upper limits to be set at annual mean values for PM10 of 15 µg/m^3^ and for PM2.5 of 5 µg/m^3^ [12].

As an individual, it is almost impossible to protect oneself from the risk factor PM10 or to completely avoid its harmful effects. Reduced exposure to PM10 may prolong life expectancy up to 7 months, if the long-term PM10 concentration is reduced by 10 µg/m^3^ [3, 13]. In particular, people with pre-existing cardiovascular or pulmonary diseases are at risk. For this reason, the "American Heart Association" recommends educating patients about the risk factor in question [3, 9].

Long-term exposure to fine dust affects the incidence of certain diseases. For example, Hoffmann et al. [3] demonstrated in their analysis of 4,433 subjects from the Ruhr area that there is a relationship between fine dust exposure and the incidence of stroke.

Furthermore, the ESCAPE study showed that the long-term exposure to PM10 is associated with an increased incidence of acute coronary events and that this relationship already exists below the EU limits [4]. Dockery et al. were able to demonstrate a statistically significant association between air pollution and mortality in an American cohort of 8,111 subjects aged between 25 and 74 years. The strongest association with a higher mortality rate was shown for the air pollution by PM10 and sulfates [5]. Similar results were seen by Pope et al. reporting an association of an increase of 10 µg/m^3^ in PM10 concentration with a 4–8% increase in mortality [6]. Heinrich et al. studied 4,800 women from North Rhine-Westphalia and included besides PM10 the concentration of NO_2_ and the distance to the nearest major road as an indirect marker of peak PM10 concentrations and noise pollution from traffic. Apparently, the proximity to a more heavily trafficked road plays a role as a risk factor [7].

This study aims to corroborate the hypothesis that residence-associated PM10 exposure has an impact on all-cause mortality and cardiovascular morbidity using data of the German Epidemiological Trial on Ankle Brachial Index (getABI) cohort over a 7-year observation period [14]. To determine whether the residence-associated NO_2_ concentration or the distance to the nearest major road influences mortality or morbidity, they were both included in the analysis. Furthermore, interactions between the established cardiovascular risk factors and environmental pollutants with regard to the selected endpoints were investigated secondarily.

## Data and methods

### Study population

The getABI study is a prospective cohort study that started in 2001 and aimed to determine the association between the ankle brachial index (ABI) and cardiovascular events in primary care patients. 34 angiology specialists recruited primary care physicians from 344 practices all over Germany. In October 2001, at least 20 patients 65 years or older per general practitioner were recruited for the study. One exclusion criterion was a life expectancy of less than 6 months. A complete health status was obtained, which included a detailed medical history with special attention to cardiovascular disease as well as medical and socioeconomic risk factors. In addition, information on height, pulse status, heart rate, blood pressure, and ABI status was gathered. Followed-up visits took place after 1, 3, 5, and 7 years. In case of death, time of death and cause of death were determined with the help of the primary care physician and registration offices [14].

### Air pollutants exposure and proximity to major road

The PM10 and NO_2_ concentrations used for the analysis are interpolated values, which are derived from the data set and the concentration prediction models of the study "Mapping of background air pollution at a fine spatial scale across the European Union" [15]. In this study geostatistical methods were used to interpolate measured data from stations in the vicinity to get an estimation for locations where no measured data were available. With help of the collected values and the universal kringing method, Europe-wide air pollution maps were generated for NO_2_ and PM10 on a 1 km^2^ grid. The data refer to the year 2001 and are derived from "Airbase", a European database with air quality data collected in the course of regular routine measurements by the EU member states, including information on land use, road traffic, population density, weather, altitude, and topography [15].

Regarding the proximity to major roads, geographers of the Ruhr University Bochum converted the places of residence of the getABI participants into Gauss-Krueger coordinates, and the distances to major roads were generated with the help of the "Functional Road Classes" from the StreetPro_EMEA program. Therein, all roads that fell under the three classes motorway, major road, and other major road (micodes 2001xxxx, 2002xxxx, and 2003xxxx, respectively) were considered together and referred to as major road in this work. A major road within a radius of ≤ 50 m to the residential address was considered an exposure to this potential risk factor.

### Definition of adjustment variables

Adjustment was performed in two a priori defined adjustment steps. Model 1 with adjustment for the variables age, sex, and one or more air pollutants formed the first step and is referred to as "basic adjustment" in the further course. Model 2 was labeled as "full adjustment" and additionally included the education status (International standard classification of eduction (ISCED)), smoking status, pack-years, body-mass-index, estimated glomerular filtration rate (eGFR), C-reactive protein (CRP), vitamin D, homocysteine, low-density lipoprotein (LDL), gamma-glutamyltransferase (GGT), arterial hypertension, diabetes mellitus, PAD, cardiovascular events prior to baseline, and intake of lipid-lowering, anticoagulant, antiplatelet, or digitalis medication.

In order to investigate a possible effect of drug therapy on the impact of PM10 exposure, the intake of different drug groups was included in the analysis. Statins or fibrates were labeled as lipid-lowering medication. Anticoagulant medication was defined as the use of coumarin derivatives or heparin. Therapeutic use of acetylsalicylic acid, ticlopidine, clopidogrel, or dipyramidol was grouped under antiplatelet medication. The intake of digitalis was considered as a separate variable.

The following seven interactions were pre-specified: each single air pollutant with PAD at baseline, NO_2_ and smoking, PM10 und CRP, PM10 and anticoagulant medication, and PM10 and antiplatelet medication. Beside the interaction term, both variables of this term were always included into the model. Thus, the basic adjustment model e.g. for the last mentioned interaction contains the variables age, sex, PM10, antiplatelet medication, and the PM10-by-antiplatelet interaction.

### Outcomes

The primary outcome in this analysis was death from any cause. Secondary, we investigated the impact of air pollutants and of proximity to major roads on the combined endpoint of death from any cause and cardio-vascular morbidity. The latter was defined as a new event of myocardial infarction, apoplexy, coronary or carotid revascularization.

The third endpoint was defined as the time to the first occurrence of PAD not yet detected at baseline. This was defined as amputation, peripheral revascularization, or occurrence of necrosis/gangrene of the affected limb during the observation period. In addition, PAD was assumed, if the ABI fell below the threshold of 0.9 during the course of the study. Because the follow-up examinations were performed at fixed time points (1, 3, 5, and 7 years after baseline), and thus information for the time between the examination time points was missing, a progression curve was generated for the ABI by regression. If this curve had an intersection with the value 0.9 or if a new PAD symptom occurred, the event occurring first was used to calculate the event-free time to adequately reflect the incidence of PAD [16].

### Statistical methods

Patients’ characteristics at baseline are also presented for subgroups defined by the quartiles of PM10 exposure in the study population.

To investigate the relationship between the different exposure factors and mortality or incidence of PAD, survival times were modeled by Cox proportional hazards regression, briefly referred to as Cox regression. If multiple target events occurred in a patient during the analysis of the combined endpoint, only the event that occurred first was considered.

Missing values rarely appeared (below 2%), on continuous variables such as CRP, eGFR, vitamin D, and ABI they were randomly supplemented with data from the sample. For categorical variables, missing values were assigned to the unexposed group.

Effects were presented in the form of HR with associated 95% confidence intervals (CI). The effects of PM10 and NO_2_ were calculated per unit. One unit corresponds to an increase of the respective concentration by 10 μg/m^3^. The effect of the variable pack-years was estimated with a unit of 10 pack-years.

The significance level was set to 0.05. For interactions, the estimated HR and 95% CI on each level of the potentially modifying factor were provided. Corrections for multiple testing of hypotheses were not applied.

## Results

### Baseline characteristics

**Fig. 1:**
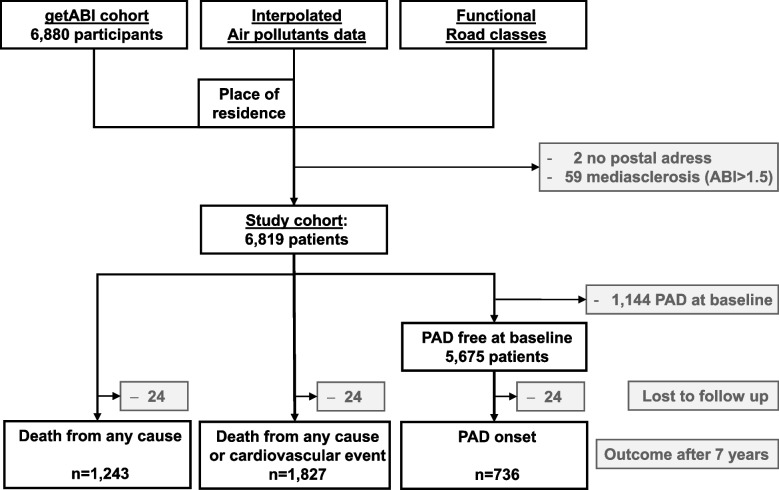
Flow chart outline of the inclusion of patients in the analyses

The getABI study recruited 6,880 patients. Two patients with a post office box as address were excluded from the analysis. In addition, 59 patients with an ABI value above 1.5 and without other PAD factors (amputation, peripheral revascularization, necrosis/gangrene) were excluded, due to assumed mediasclerosis, so that a total of 6,819 patients were included in the further analysis (Fig. 1).

For the analysis of the incidence of PAD, 1,144 patients who were already diagnosed with PAD at baseline were excluded (Fig. 1).

The maximum annual mean PM10 exposure was below the value of 40 µg/m^3^ as specified by the EU. The maximum value in the cohort was 30.88 µg/m^3^. The upper limit value of 21.45 µg/m^3^ of the first quartile was above the annual mean PM10 limit value of 15 µg/m^3^ required by the WHO, only 4 people of the cohort had a value below 15 µg/m^3^ at residence. The mean pollutant exposure to PM10 in the whole cohort at baseline was 23.34 µg/m^3^ (sd = 2.65 µg/m^3^).

More women (58.03%) than men took part in the study. Many of the 634 smokers in the study population were in the 4th quartile quarter. Patients in the quartile quarter 4 more often had elevated CRP, hyperhomocysteinemia, and an elevated serum LDL than the patients in the other groups (Table 1).

**Table 1:** Baseline patient characteristics stratified by PM10 quartiles

Baseline characteristics	ALL		Quartiles by 2001 residential annual mean of PM10 [µg/m^3^]			
	No. available	Mean (SD) or no.(%)	0–21.45 (*n* = 1,702)	> 21.45–23.48 (*n* = 1,712)	> 23.48–25.76 (*n* = 1,685)	> 25.76–30.88 (*n* = 1,720)
**Patients**						
** Age [years]**	6,819	72.53 (5.28)	72.54 (5.27)	72.37 (5.19)	72.77 (5.48)	72.45 (5.18)
** Female sex**	6,819	3,957 (58.03)	975 (57.29)	1,004 (58.64)	997 (59.17)	981 (57.03)
** Low education, ISCED 0–2**	6,819	1,697 (24.89)	506 (29.73)	404 (23.6)	366 (21.72)	421 (24.48)
** Current Smoking**	6,819	634 (9.30)	142 (8.34)	158 (9.23)	155 (9.20)	179 (10.41)
** Pack-years**	6,819	11.23 (19.95)	10.16 (19.35)	10.81 (19.22)	11.7 (20.49)	12.26 (20.65)
** BMI ≥ 30 kg/m**^**2**^	6,814	1,575 (23.10)	423 (24.85)	381 (22.25)	376 (22.31)	395 (22.97)
**Laboratory assessments**						
** eGFR ≤ 60 mL/min/m**^**2**^	6,756	1,337 (19.61)	325 (19.10)	323 (18.87)	353 (20.95)	336 (19.53)
** CRP > 3 mg/L**	6,757	2,642 (38.74)	680 (39.95)	647 (37.79)	610 (36.20)	705 (40.99)
** Vitamin D < 50 nmol/L**	6,743	4,739 (69.50)	1,172 (68.86)	1,171 (68.40)	1,175 (69.73)	1,221 (70.99)
** Homocystein > 15 µmol/L**	6,686	2,963 (43.45)	716 (42.07)	709 (41.41)	762 (45.22)	776 (45.12)
** LDL ≥ 130 mg/dL**	6,819	2,919 (42.81)	715 (42.01)	712 (41.59)	716 (42.49)	776 (45.12)
** GGT > 18 |> 26 U/L (f | m)**	6,761	1,676 (24.58)	419 (24.62)	415 (24.24)	386 (22.91)	456 (26.51)
**Pre-existing diseases**						
** Arterial Hypertension**	6,819	4,732 (69.39)	1,154 (67.80)	1,235 (72.14)	1,171 (69.50)	1,172 (68.14)
** Diabetes mellitus**	6,819	1,741 (25.53)	379 (22.27)	465 (27.16)	401 (23.80)	496 (28.84)
** PAD**	6,819	1,144 (16.78)	285 (16.75)	272 (15.89)	284 (16.85)	303 (17.62)
** Cardiovascular event**	6,819	1,091 (16.00)	236 (13.87)	264 (15.42)	287 (17.03)	304 (17.67)
**Medications**						
** Lipid lowering**	6,819	1,606 (23.55)	364 (21.39)	412 (24.07)	428 (25.40)	402 (23.37)
** Anticoagulant**	6,819	462 (6.78)	129 (7.58)	116 (6.78)	114 (6.77)	103 (5.99)
** Antiplatelet**	6,803	2,271 (33.30)	552 (32.43)	578 (33.76)	591 (35.07)	550 (31.98)
** Digitalis**	6,819	549 (8.05)	134 (7.87)	141 (8.24)	125 (7.42)	149 (8.66)
**Air pollution variables**						
** PM10 [μg/m**^**3**^**]**	6,819	23.34 (2.65)	19.82 (1.32)	22.57 (0.53)	24.26 (0.69)	26.70 (0.70)
** NO**_**2**_** [μg/m**^**3**^**]**	6,819	23.32 (6.63)	18.50 (6.01)	23.94 (6.15)	23.58 (4.89)	27.21 (6.23)
** Major Road ≤ 50 m**	6,819	379 (5.56)	96 (5.64)	96 (5.61)	114 (6.77)	73 (4.24)

Values are either mean and standard deviation for continuous variables or count and percentage for categorical variables

*PM10* Particulate matter with a diameter of 10 µm or less; *NO*_*2*_ Nitrogen dioxide; *ISCED* International standard classification of education; *BMI* Body-mass index; *eGFR* Estimated glomerular filtration rate; *CRP* C-reactive protein; *LDL* Low-density lipoprotein; *GGT* Gamma-glutamyltransferase; *PAD* peripheral artery disease

### Primary Outcome “death from any cause”

Within the study period, 1,243 patients (18.2%) died. The model with the basic adjustment with single pollution variables showed numerically increased point estimates for PM10, for NO_2_, and for distance to the nearest major road ≤ 50 m for the endpoint death of any cause. In the fully adjusted model, PM10 and the distance to the major road also showed increased but somewhat lower point estimates of HR than in the basic model and for NO_2_ a decrease for this endpoint. The associations were not statistically significant.

**Fig. 2:**
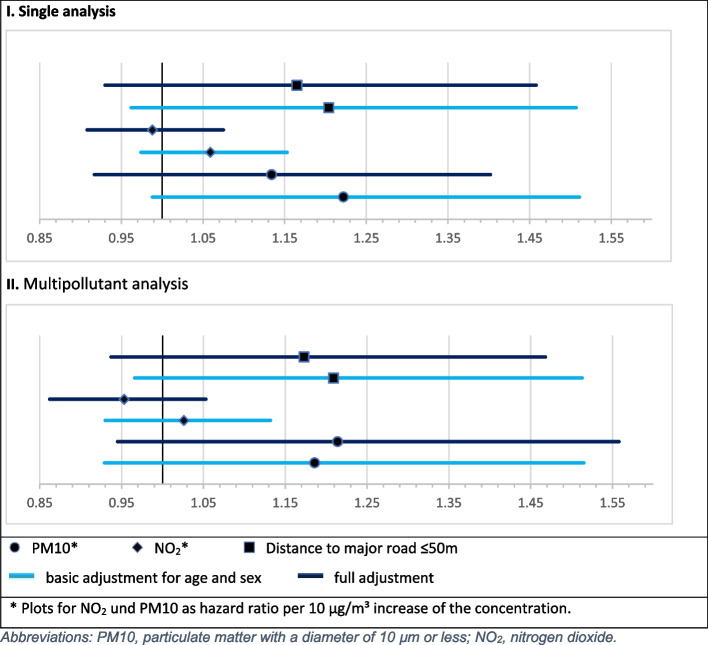
Single and multipollutant analysis of main pollutant variables. Abbreviations: PM10, particulate matter with a diameter of 10 µm or less; NO_2_, nitrogen dioxide

In the fully adjusted multipollutant approach, the risk for PM10 (HR = 1.218; 95%-CI: 0.949-1.562) was higher and for NO_2_ (HR = 0.950; 95%-CI: 0.859-1.050) slightly lower than in the above mentioned basic approach. The HR of distance to the nearest major road (HR = 1.164; 95%-CI: 0.93-1.457) remained stable. The CI of all three variables did not show statistical significance of these results (Fig. 2, Table 2).

**Table 2:** Results for the primary outcome “death from any cause”

Model			Basic adjustment	Full adjustment
			HR (95%-CI)	HR (95%-CI)
Air pollutant variables single				
PM10			1.222 (0.988; 1.511)	1.134 (0.917; 1.402)
NO_2_			1.059 (0.974; 1.153)	0.988 (0.908; 1.075)
Dist. Major Road ≤ 50 m			1.204 (0.962; 1.507)	1.165 (0.930; 1.458)
Air pollutant variables multipollutant analysis				
PM10, NO_2_, distance to Major Road ≤ 50 m	PM10		1.186 (0.929; 1.515)	1.218 (0.949; 1.562)
	NO_2_		1.026 (0.930; 1.132)	0.950 (0.859; 1.050)
	Dist. Major Road ≤ 50 m		1.209 (0.966; 1.513)	1.164 (0.930; 1.457)
Interaction terms				
Air pollutants and PAD	PM10	PAD yes	1.560 (1.059; 2.298)	1,462 (0,967; 2,209)
		PAD no	1.112 (0.863; 1.432)	1,132 (0,854; 1,502)
	NO_2_	PAD yes	1.090 (0.941; 1.263)	0,962 (0,824; 1,122)
		PAD no	1.027 (0.927; 1.138)	0,943 (0,839; 1,060)
	Dist. Major Road ≤ 50 m	PAD yes	0.957 (0.610; 1.502)	0,994 (0,632; 1,562)
		PAD no	1.324 (1.023; 1.716)	1,233 (0,951; 1,599)
NO_2_ and smoking	Smoking yes		0.989 (0.805; 1.215)	0.909 (0.735; 1.124)
	Smoking no		1.047 (0.954; 1.149)	0.958 (0.860; 1.066)
PM10 und CRP	CRP > 3 mg/L		1.304 (0.965; 1.761)	1.372 (0.986; 1.910)
	CRP ≤ 3 mg/L		1.142 (0.849; 1.536)	1.089 (0.789; 1.504)
PM10 and anticoagulants	anticoagulants yes		1.388 (0.786; 2.451)	1.343 (0.755; 2.390)
	anticoagulants no		1.226 (0.976; 1.541)	1.201 (0.924; 1.560)
PM10 and antiplatelets	antiplatelets yes		1.429 (1.021; 1.999)	1.433 (0.999; 2.056)
	antiplatelets no		1.105 (0.841; 1.453)	1.096 (0.810; 1.482)

Point estimates and 95% confidence intervals (CI) from cox proportional hazards regression with basic adjustment (age and sex, left) and full adjustment (right) additionally by ISCED, smoking status, pack-years, body-mass-index, estimated glomerular filtration rate, C-reactive protein, vitamin D, homocysteine, low-density lipoprotein, gamma-glutamyltransferase, arterial hypertension, diabetes mellitus, PAD, cardiovascular events at baseline, and lipid-lowering drug use. Units for HRs of NO_2_ and PM10 were 10 µg/m^3^ increase in concentration, and for pack-years 10

*PM10* Particulate matter with particles of size up to 10 µm; *NO*_*2*_ Nitrogen dioxide; *CRP* C-reactive protein; *PAD* Peripheral artery disease; *ISCED* International standard classification of education

PM10 exposure in combination with the presence of PAD had a significantly increased HR (HR = 1.560; 95%-CI: 1.059-2.298) for the endpoint death of any cause in the basic model and a positive point estimate in the fully adjusted model.

The risk of death increased significantly with shorter distance to the nearest major road and the absence of PAD (HR = 1.324; 95%-CI: 1.023-1.716). This association did not remain significant in the fully adjusted model.

For an elevated CRP value, the point estimate of PM10 (HR = 1.372; 95%-CI: 0.986-1.910) was higher than for a normal CRP value (HR = 1.089; 95%-CI: 0.789-1.504) for the endpoint death of any cause in the fully adjusted model. Risks for these interaction terms showed similar estimates in the basic model.

Lastly, the risk for the endpoint death from any cause was significantly increased by 43% per 10 µg/m^3^ increase of PM10, if antiplatelet medication was taken (HR = 1.429; 95%-CI: 1.021-1.999, basic adjustment). Similar point estimates were not significant in the fully adjusted model.

### Secondary Outcomes

#### Death or cardiovascular event

Until the end of the study, 1,827 patients (26.8%) had a cardiovascular event and/or died. In the model with basic adjustment, increased HR could be detected for PM10 (HR = 1.225; 95%-CI: 1.03-1.458), NO_2_ (HR = 1.102; 95%-CI: 1.029-1.181), and distance to major road ≤ 50 m (HR = 1.084; 95%-CI: 0.891-1.317), with PM10 and NO_2_ showing statistical significance. Considering the pollutant variables together in the basic model, there was still an increased point estimate, but without statistical significance for PM10 and NO_2_ (Table 3).

**Table 3:** Results for the secondary combined endpoint “death from any cause or cardiovascular event”

Model			Basic adjustment	Full adjustment
			HR (95%-CI)	HR (95%-CI)
Air pollutant variables single				
PM10			1.225 (1.030; 1.458)	1.130 (0.950; 1.345)
NO_2_			1.102 (1.029; 1.181)	1.038 (0.969; 1.112)
Dist. Major Road ≤ 50 m			1.084 (0.891; 1.317)	1.051 (0.864; 1.279)
Air pollutant variables multipollutant analysis				
PM10, NO_2_, distance to Major Road ≤ 50 m	PM10		1.116 (0.913; 1.363)	1.106 (0.902; 1.357)
	NO_2_		1.080 (0.997; 1.169)	1.017 (0.938; 1.103)
	Dist. Major Road ≤ 50 m		1.094 (0.900; 1.330)	1.057 (0.869; 1.285)
Interaction terms				
Air pollutants and PAD	PM10_2_	PAD yes	1.249 (0.902; 1.731)	1,062 (0,750; 1,504)
		PAD no	1.211 (0.986; 1.487)	1,123 (0,893; 1,412)
	NO_2_	PAD yes	1.092 (0.964; 1.237)	0,993 (0,872; 1,132)
		PAD no	1.089 (1.003; 1.182)	1,028 (0,937; 1,129)
	Dist. Major Road ≤ 50 m	PAD yes	0.823 (0.551; 1.230)	0,853 (0,570; 1,276)
		PAD no	1.187 (0.949; 1.484)	1,139 (0,911; 1,426)
NO_2_ and smoking	Smoking yes		0.963 (0.808; 1.149)	0.933 (0.781; 1.115)
	Smoking no		1.067 (0.989; 1.150)	1.034 (0.949; 1.128)
PM10 und CRP	CRP > 3 mg/L		1.215 (0.945; 1.561)	1.063 (0.820; 1.379)
	CRP ≤ 3 mg/L		1.227 (0.966; 1.559)	1.097 (0.846; 1.422)
PM10 and anticoagulants	anticoagulants yes		1.346 (0.812; 2.230)	1.146 (0.691; 1.901)
	anticoagulants no		1.230 (1.023; 1.480)	1.101 (0.890; 1.362)
PM10 and antiplatelets	antiplatelets yes		1.444 (1.104; 1.889)	1.271 (0.951; 1.699)
	antiplatelets no		1.102 (0.877; 1.385)	1.003 (0.781; 1.288)

Cox regression and interaction terms for the combined endpoint of death or cardiovascular event. Point estimates and 95% confidence intervals (CI) from cox proportional hazards regression with basic adjustment (age and sex, left) and full adjustment (right) additionally by ISCED, smoking status, pack-years, body-mass-index, estimated glomerular filtration rate, C-reactive protein, vitamin D, homocysteine, low-density lipoprotein, gamma-glutamyltransferase, arterial hypertension, diabetes mellitus, PAD, cardiovascular events at baseline, and lipid-lowering drug use. Units for HRs of NO_2_ and PM10 were 10 µg/m^3^ increase in concentration, and for pack-years 10

*PM10* Particulate matter with particles of size up to 10 µm; *NO*_*2*_ Nitrogen dioxide; *ISCED* International standard classification of education; *PAD* Peripheral artery disease

PAD in combination with the increase in PM10 exposure was associated with an increase in risk, which was also attenuated in the fully adjusted model. NO_2_ exposure was associated with a significant, slightly increased risk (HR = 1.089, 95%-CI: 1.003-1.182) in the basic model in the absence of PAD, but was no longer significant in the fully adjusted model. In the absence of PAD, an increase in the point estimate for the risk of this combined endpoint was noted in combination with a distance to the nearest major road ≤ 50 m (HR = 1.187; 95%-CI: 0.949-1.484). This was also shown to be slightly decreased in the fully adjusted model.

In the basic adjustment model, increased risk (HR = 1.444; 95%-CI: 1.104-1.889) could be noted in the presence of antiplatelet medication in combination with PM10 exposure, relatively to the PM10 risk (HR = 1.231; 95%-CI: 0.996-1.592) in the basic adjustment model supplemented by the effect for antiplatelet medication, but without an interaction term. Comparable differences of lower magnitude were also found for the interaction of PM10 and anticoagulant medication.

#### Onset of peripheral artery disease

During the cause of the study, 736 (10.8%) patients developed PAD.

In the model with the basic adjustment, slightly increased HR were observed for NO_2_ and distance to the nearest major road, which were no longer increased in the fully adjusted model (Table 4). PM10 showed a lower point estimate in the basic model, which dropped even further in the fully adjusted model.

**Table 4:** Results for the secondary outcome “onset of PAD”

Model		Basic adjustment	Full adjustment
		HR (95%-CI)	HR (95%-CI)
Air pollutant variables single			
PM10		0.966 (0.757; 1.309)	0.896 (0.681; 1.179)
NO_2_		1.037 (0.929; 1.157)	0.985 (0.881; 1.101)
Dist. Major Road ≤ 50 m		1.034 (0.754; 1.417)	0.986 (0.719; 1.353)
Air pollutant variables multipollutant analysis			
PM10, NO_2_, Dist. to Major Road ≤ 50 m	PM10	0.937 (0.683; 1.286)	0.884 (0.643; 1.217)
	NO_2_	1.050 (0.926; 1.192)	1.010 (0.888; 1.149)
	Dist. Major Road ≤ 50 m	1.036 (0.756; 1.421)	0.985 (0.718; 1.352)
Interaction terms			
NO_2_ and smoking	Smoking yes	0.835 (0.628; 1.109)	0.842 (0.629; 1.126)
	Smoking no	1.050 (0.931; 1.183)	1.045 (0.912; 1.198)
PM10 und CRP	CRP > 3 mg/L	0.948 (0.632; 1.423)	0.859 (0.551; 1.339)
	CRP ≤ 3 mg/L	1.032 (0.714; 1.493)	0.908 (0.610; 1.351)
PM10 and anticoagulants	anticoagulants yes	1.410 (0.419; 4.741)	1.231 (0.350; 4.332)
	anticoagulants no	0.976 (0.737; 1.292)	0.873 (0.631; 1.206)
PM10 and antiplatelets	antiplatelets yes	0.901 (0.568; 1.429)	0.795 (0.488; 1.295)
	antiplatelets no	1.063 (0.756; 1.493)	0.939 (0.645; 1.365)

Cox regression and interaction terms for the combined endpoint of death or cardiovascular event. Point estimates and 95% confidence intervals from cox proportional hazards regression with basic adjustment (age and sex, left) and full adjustment (right) additionally by ISCED, smoking status, pack-years, body-mass-index, estimated glomerular filtration rate, C-reactive protein, vitamin D, homocysteine, low-density lipoprotein, gamma-glutamyltransferase, arterial hypertension, diabetes mellitus, PAD, cardiovascular events at baseline, and lipid-lowering drug use. Units for HRs of NO_2_ and PM10 were 10 µg/m^3^ increase in concentration, and for pack-years 10

*PM10* Particulate matter with particles of size up to 10 µm; *NO*_*2*_ Nitrogen dioxide; *ISCED* International standard classification of education; *PAD* Peripheral artery disease

In the multipollutant analysis, the HR of the three main variables were similar to the single analysis.

In patients who were treated with anticoagulant drugs and simultaneously exposed to PM10 an increased point estimate was observed in both the basic and the fully adjusted model. The absence of anticoagulant therapy in combination with increased PM10 exposure showed point estimates below 1 in both models.

The interaction of antiplatelet medication and PM10 exposure yielded no specific findings for the risk of PAD onset.

## Discussion

This work found some hints for the impact of air pollutants (PM10, NO_2_, and proximity to major road) on mortality and cardiovascular morbidity. Furthermore, interactions of PAD with PM10, and antiplatelet medication with PM10 were found.

### Particulate matter

In 2019, the annual average PM10 value in Germany fell below 20 µg/m^3^ for the first time since measurements were recorded. Nevertheless, the value is still above the WHO recommended limit of 15 µg/m^3^. Overall, PM10 concentration values have been falling continuously since 2003 (2003 value 37 µg/m^3^) [17]. The decreasing PM10 concentrations could possibly have resulted in a lack of significance in our results. This would explain why other studies, as well as our analysis, present the effects previously described in the literature as limited, non-significant or lower in magnitude. For example, Heinrich et al. describe a decrease in PM10 levels over the course of observation and a concomitant decrease in overall and cardiovascular mortality in their 1990 subcohort compared to the 1985 subcohort.

An effect of long-term PM10 exposure on mortality, although lower than expected, cannot be dismissed. Several articles show a strong association of PM10 exposure with the incidence of stroke and an increase in the intima thickness of the carotid artery [3, 18–20]. Conflicting results with respect to the incidence of coronary events were reported from China [21]. A recent meta-analysis showed a small effect of PM10 on all-cause mortality (risk ratio (RR) 1.04, 95%-CI 1.03-1.06 per 10 µg/m^3^ increase in PM10 concentration). The prediction intervals in this analysis for the PM10 effect on cause-specific mortality (e.g. ischemic heart disease, stroke, COPD, lung cancer) showed no significance. The effects of PM2.5 were more pronounced [22]. These effects of long-term PM2.5 exposure on cause-specific mortality was confirmed in another meta-analysis [23].

Not only long-term PM exposure, but also acute increases in particulate matter concentration are associated with cardiovascular hospitalisation or death with an increase of 1.63% (95% CI 1.20-2.07) per 10 μg/m^3^ PM10 and even 2.12% for PM2.5. This increase was most prominent on the day of exposure [24].

It is possible that PM10 has an additive effect in combination with other cardiovascular risk factors such as hypertension or diabetes mellitus. Data on the association between PM10 and the incidence of PAD are incomplete, but the results of Hoffmann et al. [3] are consistent with our observation. There seems to be no clear increase of PAD incidence in the context of long-term fine dust exposure. The impact of classical risk factors such as smoking, arterial hypertension, diabetes mellitus, and a previous cardiovascular event predominate.

### Nitrogen dioxide

In 2019, an annual average value for traffic-related areas of 35 µg/m^3^ was recorded for NO_2_ in Germany. In 2017, an annual NO_2_ pollution of 40 µg/m^3^ was measured, which corresponds to the annual limit value for NO_2_ of the EU. Since road traffic is considered to be the main source of NO_2_, it is not surprising that in rural areas the values are significantly lower. In 1995, values of 53 µg/m^3^ were recorded and a minimal annual decrease could be seen over the last 25 years. Nevertheless, the decrease is small and cannot be compared with the reduction of PM10 pollution [25]. Heinrich et al. could hardly detect a reduction of NO_2_ levels in their annual follow-up measurements despite a decrease of PM10 concentrations [7]. Only in the basic adjustment model, a significant risk increase could be detected for the combined endpoint. In contrast, the current literature suggests an amplifying effect of NO_2_ on the risk of death.

By adjustment for PM10 and the distance to the nearest major road, the low effect of NO_2_ was further attenuated. It is possible that the concentrations of other air pollutants may influence the effect of NO_2_. The correlations with PM10, PM2.5 and black carbon described in the studies, could be a hint for this hypothesis and justify, why NO_2_ itself causes some difficulties. Therefore in several long-term studies, the effects could not be attributed exclusively to NO_2_ concentration, but rather to a mixture of traffic-related air pollutants. For this reason, the long-term effects of NO_2_ exposure should always be determined together with PM10 (ultrafine particles) and the air content of black carbon. The relationship between the incidence of cardiovascular disease as well as the incidence of PAD and long-term exposure to NO_2_ can only be answered with more consistent results, as the existing data do not allow a conclusion on this topic.

### Distance to major road

Several studies described a significant decrease in PM2.5, ultrafine particles, black carbon, and NO_2_ within the first 100 to 150 m distance from the nearest major road [26–28]. A small distance to a major road means exposure to a mixture of air pollutants, traffic noise, and miscellaneous pollutants from road abrasion, tire wear, and brake wear. The concentrations of carbon monoxides and polycyclic aromatic hydrocarbons, as well as various metals are elevated near major roads. These factors play a role when considering the "distance to nearest major road" as a risk factor and may be responsible for the health effects. While the influence of proximity to the nearest major road on increased mortality is reflected in many papers, evidence regarding the impact of proximity to heavy traffic on atherosclerosis, the incidence of PAD, the intimal thickness of the carotid artery, and the incidence of strokes and heart attacks is limited. Our results do not add significant information on this topic, since results are not conclusive. The work of Beelen et al. emphasizes that in addition to the distance to the major road, the traffic intensity on this road also plays a role in the risk stratification [29].

The strength of our study lies in the cohort of unselected patients from all regions in Germany and the consideration of environmental pollutants, combining the influence of long-term background exposure and likely short-term peak exposure in the vicinity of heavy traffic. Our analysis faces some limitations. The used pollutant exposure refers to the start of the getABI trial. Trends in pollutant values before and after 2001 were ignored. The interpolated pollution data carries a regression to the mean effect, so the spread in values might be underestimated. No data on PM2.5 was considered. Thus, the data compilation has weaknesses, which might have influenced the results, but other options were not available.

## Conclusions

Our analysis provides some hints for the impact of air pollutants (PM10, NO_2,_ and proximity to major road) on mortality. Interactions of PAD and PM10 as well as antiplatelets and PM10 were found. No association of air pollutants and the onset of PAD was seen.

## Data Availability

Request for sharing the trial data can be addressed to the corresponding author and will be considered on an individual basis by the authors.

## Abbreviations

CI: Confidence interval
CRP: C-reactive protein
eGFR: Estimated glomerular filtration rate
ESCAPE: European Study of Cohorts for Air Pollution Effects
getABI: German Epidemiological Trial on Ankle Brachial Index
GGT: Gamma-glutamyltransferase
HR: Hazard ratio
ISCED: International standard classification of eduction
LDL: Low-density lipoprotein
NO_2_: Nitrogen dioxide
PAD: Peripheral artery disease
PM10: Particulate matter
WHO: World Health Organization
